# Prognostic Value of Inflammatory Indices and the Advanced Lung Cancer Inflammation Index (ALI) for Survival Outcomes in Hemodialysis Patients †

**DOI:** 10.3390/medicina62091670

**Published:** 2026-08-31

**Authors:** Demet Yavuz, Mehmet Derya Demirag, Enis Akca, Muhammed Ali Atasever, Mehmet Omer Bostanci, Nihal Aydemir

**Affiliations:** 1 Department of Nephrology, Samsun City Hospital, 55080 Samsun, Türkiye; demetdolu@hotmail.com (D.Y.); nihalaydemir7677@gmail.com (N.A.); 2 Department of Internal Medicine, Samsun City Hospital, 55080 Samsun, Türkiye; mehmetderyademirag@yahoo.com; 3 Department of Oncology, Ministry of Health Basaksehir Cam and Sakura City Hospital, Basaksehir, 34480 Istanbul, Türkiye; m.aliatasever05@gmail.com; 4 Department of Physiology, Faculty of Medicine, Hitit University, 19040 Corum, Türkiye; mobostanci@hitit.edu.tr

**Keywords:** systemic immune-inflammation index, plasma atherogenic index, advanced lung cancer inflammation index, neutrophil-to-lymphocyte ratio, hemodialysis

## Abstract

*Background and Objectives*: Chronic systemic inflammation and malnutrition drive adverse outcomes in maintenance hemodialysis (HD) patients. This study investigated the prognostic utility of four accessible inflammatory indices—systemic immune-inflammation index (SII), neutrophil-to-lymphocyte ratio (NLR), atherogenic index of plasma (AIP), and advanced lung cancer inflammation index (ALI)—in predicting survival. *Materials and Methods*: This retrospective cohort study evaluated electronic health records of 222 incident HD patients in Turkey (2014–2021), followed through December 2024. Baseline biomarkers were calculated prior to HD initiation. Survival outcomes were assessed using Kaplan–Meier curves, and independent predictors of all-cause mortality were determined via multivariable Cox proportional hazards regression models. *Results*: During a median follow-up of 24.9 months, 106 patients (47.7%) died. Non-survivors presented with significantly higher median SII, NLR, and AIP, and markedly lower ALI than survivors (all *p* < 0.001). Multivariable analysis revealed that elevated SII (HR: 1.035, 95% CI: 1.020–1.050; *p* < 0.001) and NLR (HR: 1.081, 95% CI: 1.042–1.122; *p* < 0.001) were risk factors for mortality. In contrast, higher ALI demonstrated a significant protective effect on survival (HR: 0.975, 95% CI: 0.953–0.998; *p* = 0.036). Kaplan–Meier analyses confirmed superior overall survival in patients with lower SII, NLR, AIP, and higher ALI. *Conclusions*: SII, NLR, AIP, and ALI are practical, cost-effective biomarkers for mortality risk stratification in HD patients. ALI, integrating both nutritional and inflammatory dimensions, demonstrates particularly robust prognostic value for predicting survival.

## 1. Introduction

In patients with chronic kidney disease (CKD), cardiovascular disease is a major cause of mortality and plays a significant role in adverse long-term outcomes [1]. Those on maintenance hemodialysis (HD) face ongoing low-grade systemic inflammation, a condition strongly tied to heightened morbidity and mortality [2]. Beyond its cardiovascular consequences, persistent inflammatory activity also promotes protein-energy wasting (PEW), a key predictor of unfavorable outcomes in this population [2,3]. Taken together, assessing both inflammatory burden and nutritional status may offer important advantages for risk stratification and therapeutic decision-making in HD patients [4].

Hematological indices that reflect inflammatory activity have gained increasing attention as candidate prognostic tools in CKD. The systemic immune-inflammation index (SII) has been shown to correlate with all-cause mortality in this setting [5], while elevated SII and neutrophil-to-lymphocyte ratio (NLR) have both been linked to worse survival [5,6]. Derived entirely from routine blood tests, these markers are cost-effective and readily applicable in clinical practice for gauging systemic inflammatory load and detecting patients at elevated risk [5,6].

The atherogenic index of plasma (AIP), calculated as the logarithmic ratio of triglycerides (TG) to high-density lipoprotein cholesterol (HDL-C), has been investigated in multiple populations as a marker of atherosclerosis and cardiovascular risk [7,8]. In dialysis populations specifically, AIP has shown a meaningful relationship with both mortality rates and overall survival [9,10].

Originally proposed as a prognostic tool in metastatic non-small cell lung cancer, the advanced lung cancer inflammation index (ALI) incorporates BMI, serum albumin, and NLR into a single composite score [11]. More recently, ALI has been linked to cardiovascular and overall mortality across a range of chronic conditions, including hypertension, heart failure, and peritoneal dialysis [12,13,14].

Despite individual investigations into each of these markers, data on their combined prognostic utility specifically within maintenance hemodialysis populations remain scarce. The present study therefore examined the relationships between SII, NLR, AIP, and ALI and all-cause mortality in patients undergoing maintenance hemodialysis [15].

## 2. Methods

This retrospective cohort study enrolled patients who initiated treatment at the Hemodialysis Unit of Samsun University Hospital between January 2014 and January 2021, with follow-up continuing until December 2024. The study was conducted in accordance with the Declaration of Helsinki and approved by the Non-Interventional Clinical Research Ethics Committee of Samsun University Faculty of Medicine (protocol no: GOKAEK 2025/22/5). Clinical information and ICD-10 diagnostic codes (available upon request) were obtained from electronic hospital databases and archived medical records, with investigators having full access to the EHR dataset. Data cleaning involved resolving duplicate entries and excluding incomplete records. Due to the retrospective nature of the study, the requirement for informed consent was waived by the ethics committee.

Exclusion criteria encompassed patients under 18 years of age, prior kidney transplant recipients, those transitioned from peritoneal dialysis, individuals transferred from other dialysis facilities after HD initiation, and those with fewer than six months of maintenance HD. Patients lacking baseline fasting glucose or triglyceride values were similarly excluded, as were those with active or chronic infection, liver cirrhosis, or chronic obstructive pulmonary disease. Following application of these criteria, a total of 222 patients were included in the final analysis.

Patients who had been receiving dialysis treatment at our hemodialysis unit for at least six months were included in the study. The initial blood values recorded for these patients at the end of the six-month period were taken into account. These blood values and laboratory data constituted the primary variables examined. Clinical and anthropometric characteristics, including age, sex, BMI, dialysis duration, and comorbid conditions (hypertension and diabetes mellitus), were sourced from the hospital information system and patient records.

Pre-HD laboratory parameters included serum urea, creatinine, albumin, hemoglobin, white blood cell count, platelet count, lymphocyte and neutrophil counts, ferritin, triglyceride, fasting blood glucose, total cholesterol, LDL-C, and HDL-C levels.

The following formulas were used to calculate each index:

- SII = Platelets × Neutrophils/Lymphocytes [5,6].
- NLR = Neutrophils/Lymphocytes [5,6].
- AIP = log [TG/HDL-C] [7].
- ALI = BMI (kg/m^2^) × Albumin (g/dL)/NLR [11].

BMI was calculated by division of body weight in kilograms by height in meters squared.

For the analyses, median values were calculated for each index, and patients were categorized into low and high groups according to the median cutoff values, as follows:

- SII: low SII < 653.79; high SII ≥ 653.79.
- ALI: low ALI < 24.82; high ALI ≥ 24.82.
- NLR: low NLR < 3.37; high NLR ≥ 3.37.
- AIP: low AIP < 0.66; high AIP ≥ 0.66.

All-cause mortality served as the primary study endpoint. Patients were censored upon kidney transplantation, switching to peritoneal dialysis, referral to another center, or when contact could no longer be maintained. A diagnosis of hypertension required either two separate resting blood pressure readings of ≥140 mmHg systolic and/or ≥90 mmHg diastolic, or ongoing antihypertensive therapy [16]. Type 2 diabetes mellitus was identified and managed in line with the standards set forth by the American Diabetes Association (ADA) [17]. Cardiovascular and cerebrovascular disease was defined as follows: clinically defined by the presence of a history of angina, myocardial infarction, arrhythmia, angioplasty, coronary artery bypass grafting, heart failure, peripheral vascular disease, and stroke.

### Statistical Analysis

All statistical analyses were performed using IBM SPSS Statistics for Windows, Version 26.0 (IBM Corp., Armonk, NY, USA). Categorical variables were reported as frequencies and percentages [n (%)]. Continuous variables were described as mean ± standard deviation (SD) when normally distributed, and as median with range (minimum–maximum) when distributions were skewed. Group comparisons for categorical data were performed using the chi-square test or Fisher’s exact test, depending on expected cell counts. For continuous variables, Student’s *t*-test was applied for normally distributed data, while the Mann–Whitney U test was used when normality assumptions were not met. Independent predictors of mortality were determined using multivariable Cox proportional hazards regression analysis. Survival outcomes were evaluated using Kaplan–Meier analysis, and differences between survival curves were assessed with the log-rank test. Median survival estimates are reported with standard error (SE).

## 3. Results

Over the ten-year observation period, 250 patients commenced maintenance hemodialysis at our center. After applying the eligibility criteria, 222 patients entered the final analysis. The number of patients who started treatment at our hemodialysis unit over a 10-year period was 250. Of these 250 patients, 4 switched to peritoneal dialysis, 4 underwent kidney transplantation, and 8 transferred to another center; additionally, 8 patients developed acute infections, while 2 developed chronic infections and 2 developed malignancies, resulting in their exclusion from the study. Median follow-up was 24.9 months (range 8.1–120 months). By the end of follow-up, 116 patients (52.3%) were alive, and 106 (47.7%) had died.

Mean age did not differ significantly between survivors and non-survivors (54.10 ± 13.49 vs. 56.22 ± 13.59 years; *p* = 0.54). The cohort included 120 women (54.1%) and 102 men (45.9%), with male sex being disproportionately represented among those who died (*p* < 0.01).

Survivors had significantly longer dialysis duration than non-survivors [41.3 (7–120) vs. 29.1 (8.1–119) months; *p* < 0.001] and higher BMI (26.33 ± 4.69 vs. 23.04 ± 3.28 kg/m^2^; *p* < 0.001). Hypertension, diabetes mellitus, cardiovascular disease, and cerebrovascular disease were all more prevalent in the non-survivor group (all *p* < 0.05).

Deceased patients had significantly greater white blood cell, neutrophil, and platelet counts relative to survivors (all *p* < 0.001), whereas lymphocyte counts showed no meaningful difference between the two groups (*p* = 0.69). Total cholesterol and HDL-C concentrations were similar across both groups; however, non-survivors exhibited notably higher LDL cholesterol and triglyceride levels [LDL: 99 (40–224) vs. 87 (22–224) mg/dL, *p* < 0.01; triglycerides: 179 (60–390) vs. 141.5 (39–490) mg/dL, *p* < 0.001]. A comprehensive overview of baseline demographic, clinical, and laboratory data is provided in Table 1.

Regarding the baseline inflammatory and nutritional indices, non-survivors presented with significantly higher median SII, NLR, and AIP values, and markedly lower ALI values than survivors (all *p* < 0.001; Table 2).

To determine which inflammatory markers independently predicted mortality, separate multivariable Cox regression analyses were carried out, incorporating sex, hypertension, diabetes mellitus, cardiovascular disease, cerebrovascular disease, and the relevant index as covariates in each model. Cox regression analysis was conducted by including dialysis duration, BMI, creatinine, albumin, LDL, and triglycerides in the model for surviving and deceased patients. SII (HR: 1.035, 95% CI: 1.020–1.050; *p* < 0.001), NLR (HR: 1.081, 95% CI: 1.042–1.122; *p* < 0.001), and AIP (HR: 2.656, 95% CI: 0.416–16.963; *p* = 0.302), ALI (HR: 0.975, 95% CI: 0.953–0.998; *p* = 0.036) The results of the multivariable Cox regression analysis are presented in Table 3.

**Figure 1:**
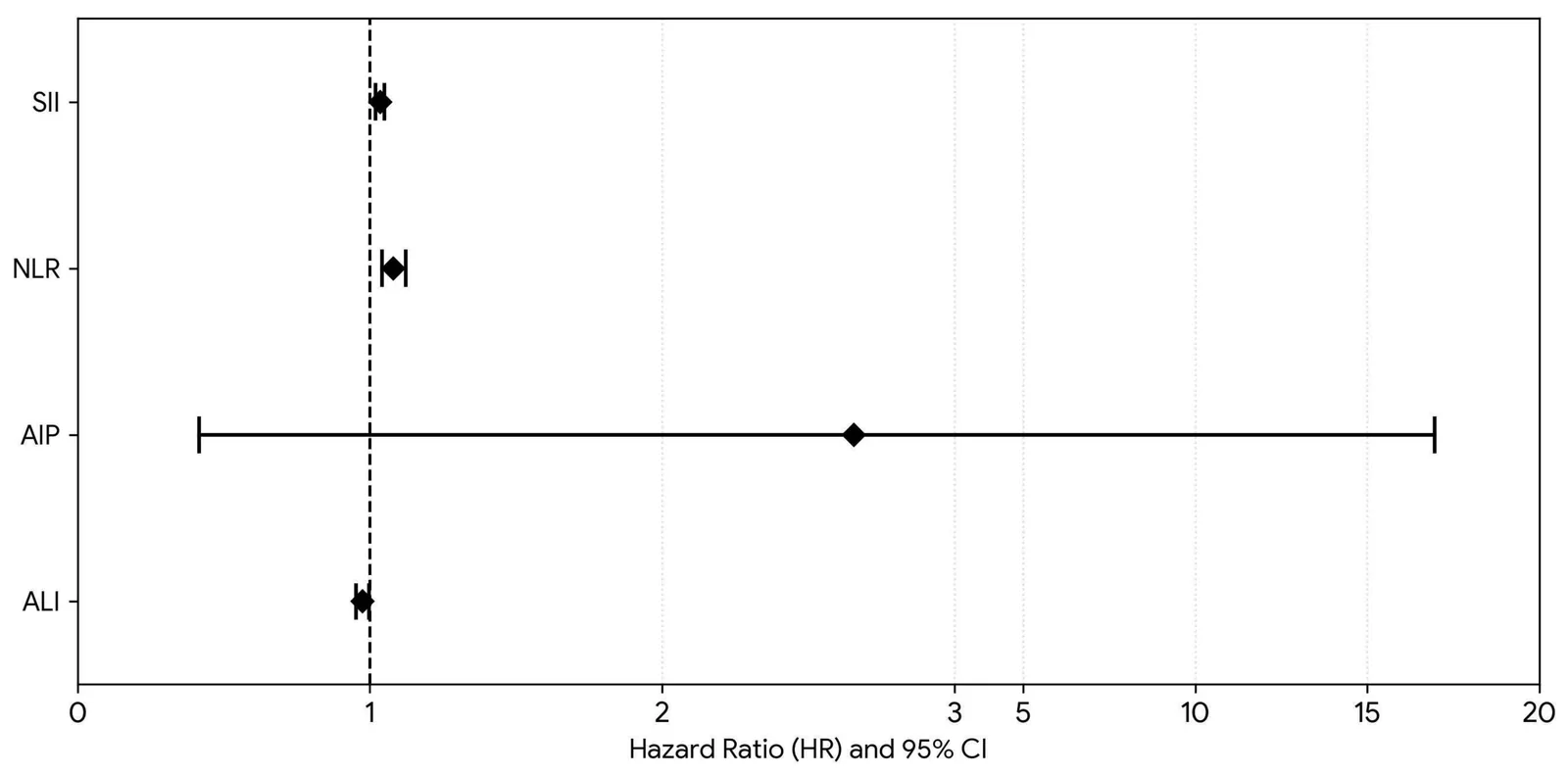
Forest plot showing mortality rates for each index included in the study. The diamonds represent the hazard ratio (HR) point estimates, and the horizontal lines indicate the 95% confidence intervals (CI). The dashed vertical line represents the line of no effect (HR = 1).

A forest plot regarding mortality was generated for each index (SII, NLR, AIP, ALI) (Figure 1).

### 3.1. SII and Mortality

**Figure 2:**
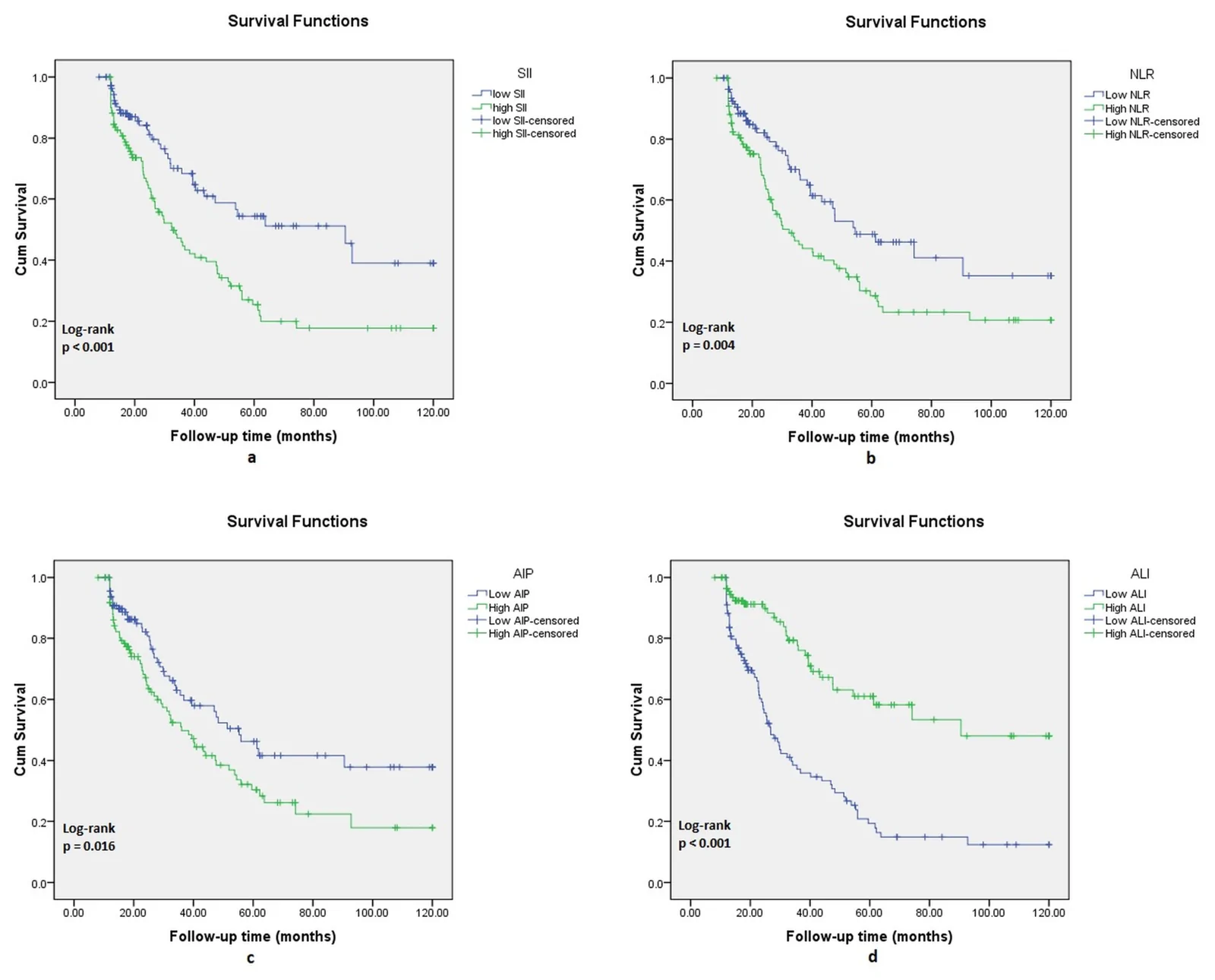
Kaplan–Meier survival curves for all-cause mortality in maintenance hemodialysis patients stratified by baseline inflammatory indices. Patients were divided into high” and “low” groups based on the median values of each respective index. (**a**) Survival analysis according to the systemic immune-inflammation index (SII) (median cutoff: 653.79). (**b**) Survival analysis according to the neutrophil-to-lymphocyte ratio (NLR) (median cutoff: 3.37). (**c**) Survival analysis according to the atherogenic index of plasma (AIP) (median cutoff: 0.66). (**d**) Survival analysis according to the advanced lung cancer inflammation index (ALI) (median cutoff: 24.82). Differences between survival curves were assessed using the log-rank test. Tick marks indicate censored patients.

Deaths occurred in 35 patients (31.5%) in the low-SII group and 71 patients (63.9%) in the high-SII group. Median survival was substantially longer in those with lower SII (90.5 ± 17.9 vs. 32.4 ± 3.79 months), and Kaplan–Meier analysis confirmed a significant survival advantage in the low-SII group (log-rank *p* < 0.001) (Figure 2a).

### 3.2. NLR and Mortality

Death occurred in 39 patients (35.1%) with low NLR and 67 patients (60.4%) with high NLR. Median survival was 54.5 ± 12.3 months versus 32.3 ± 4.2 months, respectively, with Kaplan–Meier analysis confirming superior survival in the low-NLR group (log-rank *p* = 0.004) (Figure 2b).

### 3.3. AIP and Mortality

Among patients with low AIP, 42 (37.8%) died during follow-up, compared with 64 (57.7%) in the high-AIP group. Median survival was longer in the low-AIP group (55.3 ± 7.41 vs. 36.0 ± 4.2 months), with Kaplan–Meier analysis showing a statistically significant survival benefit (log-rank *p* = 0.016) (Figure 2c).

### 3.4. ALI and Mortality

Death was recorded in 76 patients (68.5%) with low ALI versus 30 patients (27.0%) with high ALI. Median survival was markedly shorter in the low-ALI group (26.8 ± 2.4 vs. 90.6 ± 12.82 months), and Kaplan–Meier analysis demonstrated significantly worse survival outcomes for these patients (log-rank *p* < 0.001) (Figure 2d).

## 4. Discussion

Our results in the present study indicate that higher ALI combined with lower SII, NLR, and AIP values were each independently linked to greater survival in patients on maintenance hemodialysis.

Male sex and the presence of comorbidities, notably hypertension, diabetes mellitus, and cardiovascular and cerebrovascular disease, were more common in patients who did not survive, suggesting that both sex and cumulative comorbidity burden play a role in mortality risk within this population. Non-survivors also presented with shorter dialysis vintage and lower serum albumin, BMI, creatinine, and hemoglobin levels, while inflammatory indices were uniformly elevated in this group. These patterns underscore the interconnected relationship between systemic inflammation, protein-energy wasting, and adverse outcomes in HD patients. Although total and HDL cholesterol were comparable between groups, elevated LDL cholesterol and triglyceride levels in non-survivors pointed to a more unfavorable cardiovascular risk profile.

Chronic kidney disease is characterized by persistent immune dysregulation involving heightened neutrophil-driven inflammatory activity alongside disrupted lymphocyte-mediated adaptive immunity [18]. Hematological indices such as SII and NLR, derivable from standard blood tests, may therefore offer a practical indirect reflection of systemic inflammation in CKD [5]. Because low-grade chronic inflammation is a well-established contributor to both CKD progression and mortality [5], SII and NLR were selected for evaluation in the present study on account of their simplicity and routine availability. Both indices were significantly elevated among deceased patients. These findings suggest that each of the markers mentioned in our study could be used to predict mortality.

Malnutrition is prevalent in dialysis patients, and in this context relatively higher lipid concentrations may reflect preserved nutritional and metabolic reserves [19]. The concept of “reverse epidemiology” in dialysis populations suggests that lipid parameters conventionally regarded as harmful may carry different prognostic implications [20]. Some prior studies have reported that higher LDL-C, HDL-C, and triglyceride levels may be paradoxically associated with reduced mortality in dialysis patients, potentially because adequate lipid stores support immune defense and resistance to infection [21].

In the present cohort, total and HDL cholesterol did not differ between groups, yet LDL-C and triglycerides were significantly higher among non-survivors, an observation that may seem to contradict the reverse epidemiology narrative. A plausible explanation is that elevated LDL-C in this population mirrored a heavier atherosclerotic burden, compounded by greater comorbidity (particularly diabetes) and more pronounced systemic inflammation among those who died. The relatively modest sample size may also have influenced these results, and larger prospective work is warranted to clarify this relationship.

Prior studies examining the TG/HDL-C ratio in dialysis patients have found that the highest ratios correspond to significantly worse survival and elevated mortality compared with the lowest categories [22,23], though some reports have questioned whether triglyceride and HDL-C levels alone reliably function as independent prognostic markers in this setting [24,25]. Consistent with earlier findings, triglyceride levels in our cohort were significantly higher among non-survivors, although the independent prognostic contribution of triglycerides alone was not separately analyzed.

Atherogenic dyslipidemia, marked by elevated triglycerides and reduced HDL cholesterol, is recognized as a cardiovascular risk factor, particularly in individuals with hypertension [26]. The AIP, derived from the log-transformed TG-to-HDL-C ratio, is considered a composite marker of lipid-related metabolic disturbance and cardiovascular risk [27], and higher AIP values have been associated with non-obstructive coronary artery disease and worse post-myocardial infarction outcomes [28]. A prior dialysis study described a U-shaped mortality relationship with AIP, whereby both very low (≤0.20) and very high (≥0.71) values independently predicted all-cause mortality [9], and a peritoneal dialysis study similarly found that higher TG/HDL-C ratios correlated with greater mortality risk [29]. In our study, AIP levels were significantly higher in patients who died, and survival was greater in patients with low AIP values; however, the effect of AIP on survival was not statistically significant in the Cox regression analysis.

In CKD, serum albumin acts as a negative acute-phase reactant whose hepatic synthesis is suppressed by inflammatory activity, limiting its value as a stand-alone nutritional marker [30]. BMI, while commonly used to gauge nutritional status, can be confounded by fluid retention and shifts in body composition [31]. The ALI integrates serum albumin, BMI, and NLR into a single score initially validated in non-small cell lung cancer and subsequently in various other malignancies [11,32]. Beyond oncology, ALI has been reported to outperform several immunoinflammatory indices in predicting sarcopenia, and low ALI values have been associated with a roughly 2.3-fold increase in malnutrition-related mortality risk [33].

Persistent low-grade inflammation in hemodialysis patients promotes protein-energy wasting through catabolism, appetite suppression, and insulin resistance [34]. This interplay is reflected in the malnutrition–inflammation–atherosclerosis (MIA) syndrome, which encapsulates how chronic inflammation, nutritional deterioration, and accelerated atherosclerosis jointly elevate cardiovascular and overall mortality in dialysis populations [35].

Consistent with this framework, non-survivors in our study had significantly lower BMI, albumin, and ALI values, and both reduced ALI and lower BMI were independent predictors of mortality. Because ALI simultaneously captures inflammatory and nutritional dimensions, it may convey more comprehensive prognostic information than individual laboratory values in isolation. These results support ALI as a clinically accessible tool for mortality risk stratification in HD patients.

### Limitations

We have to acknowledge some limitations. The retrospective single-center design and relatively small sample size and short follow-up period introduce the possibility of selection bias and constrain generalizability. Follow-up duration may be insufficient to capture long-term outcomes fully. Analyses were restricted to baseline measurements at HD initiation, and longitudinal changes in inflammatory and nutritional parameters were not tracked. Using routine retrospective data risks misclassification, missing variables, and unmeasured residual confounding. Larger prospective multicenter studies that directly compare SII, NLR, AIP, and ALI with established tools such as the malnutrition–inflammation score (MIS), protein-energy wasting criteria, and sarcopenia assessments are needed to better define the clinical utility of these biomarkers in the HD population. One of the limitations of our results is that the cut-off values for the indices we used were determined based on the median. We recommend that this point be taken into consideration when interpreting our results.

## 5. Conclusions

In summary, SII, NLR, AIP, and ALI are inexpensive and easily accessible markers that clinicians can readily use to assess the prognosis and mortality of hemodialysis patients. We believe that these markers—alongside traditional inflammation-based indices—can be utilized to predict prognosis and determine mortality risk in this patient population.

## Figures and Tables

**Table 1:** Baseline demographic, clinical, and laboratory characteristics of the study cohort according to survival status.

Variables	Patients Survived (n: 116)	Patients Deceased (n: 106)	*p*-Value
Age (years), mean ± SD	54.10 ± 13.49	56.22 ± 13.59	0.54
Gender (Male), n (%)	42 (36.2)	60 (56.6)	<0.01
Dialysis duration, med (min–max)	41.3 (7–120)	29.1 (8.1–119)	<0.001
BMI, mean ± SD	26.33 ± 4.69	23.04 ± 3.28	<0.001
Hypertension	55 (47.4)	71 (67)	<0.01
DM	47 (40.5)	81(76.4)	<0.001
Cardiac and Cerebrovascular Events	25 (21.6)	51(48.1)	<0.001
Kt/V, mean ± SD	1.48 ± 0.25	1.46 ± 0.31	0.121
Urea (mg/dL), med (min–max)	122.9 (49–238.2)	113 (7.8–367)	0.07
Creatinine (mg/dL), med (min–max)	7.61 (1.64–19.24)	4.85 (1.7–15)	<0.001
Albumin (g/dL), mean ± SD	3.71 ± 0.31	3.09 ± 0.51	<0.001
Hemoglobin (g/dL), mean ± SD	10.88 ± 1.52	9.73 ± 1.27	<0.001
WBC (×10^9^ g/L), med (min–max)	5.79 (2.74–12.08)	7.8 (2.5–29.4)	<0.001
Lymphocyte (×10^9^ g/L), med (min–max)	1.3 (0.54–2.73)	1.3 (0.2–5.4)	0.69
Neutrophil (×10^9^ g/L), med (min–max)	3.84 (1.08–9.07)	5.5 (1.3–27.1)	<0.001
PLT (×10^9^ g/L), med (min–max)	180.5 (47–351)	216 (45–690)	<0.001
Ferritin (ng/mL), med (min–max)	427.5 (35.2–1099)	417.7 (19.6–2000)	0.81
Glucose (mg/dL), med (min–max)	131.35 (67.9–665.6)	135.4 (82–565.6)	0.29
Total Cholesterol (mg/dL) mean ± SD	159.87 ± 41.11	158.07 ± 35.77	0.32
HDL (mg/dL) mean ± SD	36.75 ± 9.04	34.93 ± 7.24	0.10
LDL (mg/dL) med (min–max)	87 (22–224)	99 (40–224)	<0.01
TG (mg/dL) med (min–max)	141.5 (39–490)	179 (60–390)	<0.001

Data are presented as mean ± standard deviation (SD) for normally distributed continuous variables, median (minimum–maximum) for skewed continuous variables, and number (percentage) for categorical variables. Group comparisons were performed using Student’s *t*-test, Mann–Whitney U test, or Chi-square/Fisher’s exact test, as appropriate. Abbreviations: BMI, body mass index; DM, diabetes mellitus; WBC, white blood cell; PLT, platelets; HDL, high-density lipoprotein; LDL, low-density lipoprotein; TG, triglycerides.

**Table 2:** Comparison of baseline inflammatory and nutritional indices between surviving and deceased hemodialysis patients.

Variables	Patients Survived (n: 116)	Patients Deceased (n: 106)	*p*-Value
SII, med (min–max)	503.24 (113.82–2056.64)	841.53 (152.1–9440.5)	<0.001
NLR, med (min–max)	2.80 (0.65–7.43)	3.86 (1.3–39.5)	<0.001
AIP, med (min–max)	0.59 (0.04–1.33)	0.70 (0.23–1.11)	<0.001
ALI, med (min–max)	34.02 (10.54–137.92)	16.98 (1.37–52.79)	<0.001

Data are expressed as median (minimum–maximum). *p*-values were derived from the Mann–Whitney U test. Abbreviations: SII, systemic immune-inflammation index; NLR, neutrophil-to-lymphocyte ratio; AIP, atherogenic index of plasma; ALI, advanced lung cancer inflammation index.

**Table 3:** Multivariable Cox proportional hazards regression analysis of inflammatory indices for predicting all-cause mortality.

Variables	Hazard Ratio (HR)	95% CI	*p*-Value
SII	1.035	1.020–1.050	<0.001
NLR	1.081	1.042–1.122	<0.001
AIP	2.656	0.416–16.963	0.302
ALI	0.975	0.953–0.998	0.036

Four separate multivariable models were constructed. Each index (SII, NLR, AIP, and ALI) was entered into its respective model individually alongside established clinical covariates, including sex, hypertension, diabetes mellitus, history of cardiovascular/cerebrovascular events, BMI, creatinine level, Kt/V, albumin. Hemoglobin, low-density lipoprotein and triglyceride. Unlike other indices, the HR for SII was calculated per 100 units to demonstrate clinical significance. For this purpose, each obtained SII value was divided by 100 and included in the model. Abbreviations: HR, hazard ratio; CI, confidence interval; SII, systemic immune-inflammation index; NLR, neutrophil-to-lymphocyte ratio; AIP, atherogenic index of plasma; ALI, advanced lung cancer inflammation index.

## Data Availability

The data that support the findings of this study are available from the corresponding author, EA, upon reasonable request.

## Abbreviations

The following abbreviations are used in this manuscript:

ADA	American Diabetes Association
AIP	Atherogenic index of plasma
ALI	Advanced lung cancer inflammation index
BMI	Body mass index
CI	Confidence interval
CKD	Chronic kidney disease
DM	Diabetes mellitus
EHR	Electronic health record
HD	Hemodialysis
HDL-C	High-density lipoprotein cholesterol
HR	Hazard ratio
ICD-10	International Classification of Diseases, Tenth Revision
LDL-C	Low-density lipoprotein cholesterol
MIA	Malnutrition–inflammation–atherosclerosis
MIS	Malnutrition–inflammation score
NLR	Neutrophil-to-lymphocyte ratio
PEW	Protein-energy wasting
PLT	Platelets
SD	Standard deviation
SE	Standard error
SII	Systemic immune-inflammation index
TG	Triglycerides
WBC	White blood cell
